# Tapping the Full Potential? Jumping Performance of Volleyball Athletes in Game-Like Situations

**DOI:** 10.3389/fpsyg.2018.01375

**Published:** 2018-08-07

**Authors:** Marie-Therese Fleddermann, Karen Zentgraf

**Affiliations:** Department of Movement Science and Training in Sports, Institute of Sport Sciences, Goethe University Frankfurt, Frankfurt, Germany

**Keywords:** dual-task, cognitive-motor interference, block jumping, elite sports, perceptual-cognitive expertise, volleyball

## Abstract

**Background:** One key issue in elite interactive team sports is the simultaneous execution of motor actions (e.g., dribbling a ball) and perceptual-cognitive tasks (e.g., visually scanning the environment for action choices). In volleyball, one typical situation is to prepare and execute maximal block jumps after multiple-options decision-making and concurrent visual tracking of the ongoing game dynamics to find an optimal blocking location. Based on resource-related dual- and multi-tasking theories simultaneous execution of visual-cognitive and motor tasks may interfere with each other. Therefore, the aim of this study was to investigate whether volleyball-specific perceptual-cognitive demands (i.e., divided attention, decision making) affect blocking performance (i.e., jumping performance and length of the first step after the ready-block-position) compared to relatively isolated jumping performance.

**Methods:** Twenty-two elite volleyball players (1st – 3rd German league) performed block jumps in front of a net construction in a single-task condition (ST) and in two perceptual (-cognitive) dual-task conditions including a dual-task low (DT_L; presenting a picture of an opponent attack on a screen) and a dual-task high condition (DT_H; presenting videos of an offensive volleyball set play with a two-alternative choice).

**Results:** The results of repeated-measures ANOVAs showed a significant effect of conditions on jumping performance [*F*(2,42) = 33.64, *p* < 0.001, η_p_^2^ = 0.62] and on the length of the first step after the ready-block-position [*F*(2,42) = 7.90, *p* = 0.001, η_p_^2^ = 0.27). *Post hoc* comparisons showed that jumping performance in DT_H (*p* < 0.001) and DT_L (*p* < 0.001) was significantly lower than in ST. Also, length of the first step after the ready-block-position in DT_H (*p* = 0.005) and DT_L (*p* = 0.028) was significantly shorter than in ST.

**Conclusion:** Our findings suggest that blocking performance (i.e., jumping height, length of the first step) decreases in elite volleyball players when a perceptual (-cognitive) load is added. Based on the theory of Wickens (2002), this suggests a resource overlap between visual-processing demands for motor performance and for tracking the dynamics of the game. Interference with the consequence of dual-task related performance costs can therefore also be found in elite athletes in their specific motor expert domain.

## Introduction

In interactive team sports, athletes act in complex and dynamic environments, with the player itself, balls, teammates, opponents, referees, and sometimes the coach and the spectators moving in space with periodic changes in situational requirements such as attacking or defending (Gréhaigne et al., 2005; Lennartsson et al., 2014). In this context, perceptual-cognitive demands need to be processed concurrently to motor execution such as running, dribbling, or passing the ball. In elite volleyball, players not only have to spike or pass the ball at a specific spatial location, they also, in a preparatory manner, have to transport their bodies to the spot where the adequate technique has to be executed. Major parts of practice are allocated to improve these technical details related to anticipatory leg/foot work and ball contact skills in isolation from tactical demands (Gabbett et al., 2006). This is true for receiving, spiking, blocking, or defending (Gabbett et al., 2006; Katic et al., 2006). During competition or in game-like practice situations, however, these techniques are combined with visual-tactical requirements such as monitoring ball and opponents’ trajectories, decision-making for blocking or defending positions or for setting locations for the counterattack. One success-oriented goal for the attacking team is to “move" the opponent blockers in the wrong direction along the net, i.e., for the setter to pass the ball at a position remote from the initial position of the opponent blockers (Gasse, 1995; Gonzalez-Silva et al., 2016). Therefore, a typical situation for a blocker is to be aware of the number and position of the opponent attackers, to shortly observe the ball trajectory after reception, to position adequately for the upcoming attack by performing preparatory block steps along the net, to concurrently observe the attackers approach direction and to then timely jump maximally for reaching the hands over the net toward the ball with the aim to block the ball or at least to slow down the ball to facilitate defense by a teammate (Westphal and Gasse, 1985; Gasse, 1995; Afonso et al., 2005; Ficklin et al., 2014).

In ball sports, obviously, with its dynamic nature, execution of motor skills is inevitably linked to and needs to be adapted to perceptual-cognitive requirements. Nevertheless, expert players seem to perform these motor skills effortlessly. Fitts and Posner (1967) declared this stage as the “autonomous” stage, where movements are consistent and presumably require no or little cognitive control, so that attention may be focused on tactical choices. In the dual-task literature, a great number of studies has focused on the attentional requirements for motor and perceptual-cognitive tasks and their integration as the capacity to process several streams of information in parallel seems to be restricted (for an overview, see Woollacott and Shumway-Cook, 2002; Yogev-Seligmann et al., 2008; Al-Yahya et al., 2011; Krasovsky et al., 2017; Leone et al., 2017). Many studies suggest that some attentional resources are essential to integrate sensory (visual, vestibular, tactile, proprioceptive, acoustic, etc.) (re-)afferences and motor efferences (Dietrich, 2006; Hamacher et al., 2015; Krasovsky et al., 2017). Conceptual ideas explain performance decrements by a structural limitation of capacities (e.g., Pashler, 1994, 2000) or by limited multiple resource pools (Wickens, 2002, 2008). The multiple-resource theory, which refers to four dimensions (modalities, stages of processing, codes of processing, and response channels) postulates that predicted interference is more probable if time-shared tasks use resources from dimensions with spatially closer distances.

To understand the seemingly restricted information-processing capacity needed for motor and perceptual or cognitive tasks, in the dual-task literature single and dual-task conditions are used. For example, a primary motor task such as walking or balancing is analyzed when it is either performed as a single task (ST) or when a concurrent secondary task such as serial subtraction, letter-saying, or a reaction time go/no-go task (Beauchet et al., 2005; Beurskens et al., 2014, 2015, 2016a) is added (dual-task condition, DT). In case these two tasks compete for attentional resources within the same domain related to the modality, the stages (perception, cognition, response) or the codes, a more resource-consuming primary or secondary task should then interfere with the respective other task. Based on the specific context or personal factors such as specificity of the chosen tasks, age, familiarity with the tasks, etc., this interference may show in performance decrements, called dual-task costs. In the motor domain, performance outcome as well as production measures (Magill, 2004) have been used to quantify these changes in motor behavior. Some studies exhibited a reduction in gait velocity in DT in children (Beurskens et al., 2015), adults (Mirelman et al., 2014), and seniors (Doi et al., 2013), higher spatiotemporal gait variability in seniors in DT (Beurskens and Bock, 2012) and adults (Mirelman et al., 2014) or an increased number of missteps in seniors (Schrodt et al., 2004).

Dietrich (2006) proposed that reduced gait speed and increased gait variability in DT is due to brain-metabolism demands: integrating gait-related sensory input and motor output plus an extra perceptual-cognitive task may exceed the brain’s resources. Also, other studies (Beurskens et al., 2014; Mirelman et al., 2014) investigated cognitive-motor interference on a neurophysiological level (e.g., fNIRS) and showed increased neural activation in a dual-task paradigm. Beurskens et al. (2016c) postulated an increased cognitive load and that upregulated brain activity compensates for dual-task requirements.

Tucker and Stern’s (2011) cognitive-reserve theory suggests that individuals differ in their cognitive capacity that allows for situational compensation via the recruitment of additional brain regions and that cognitive capacity is malleable via training interventions. This might be one explanation why other studies do not show any interference between motor and cognitive tasks (Huxhold et al., 2006; Meester et al., 2014). Leone et al. (2017) also reported inconsistent findings including supra-additive activation of brain areas, but also sub-additive activation, in DT performance, presumably related to situational and differential compensation mechanisms in the participants to execute both tasks concurrently with an adequate resource allocation. These ambiguous findings for *when* interference occurs may stem from the low predictive value of the named models for *specific* DT situations. Nevertheless, when predicting the magnitude of interference between a motor task such as body transport inducing optic flow and a concurrent visually based decision-making task, the focus is on the substantial time-shared and overlapping brain resources of these two tasks. Due to this, it can be expected that also overlearned and highly repeated motor skills in elite athletes (e.g., block steps and block jumping) may still be vulnerable to secondary tasks such as concurrent tactical processing.

In addition, there are only few studies which investigated other, more sport-related movements (e.g., jumping performance). Also, there is no study which investigated cognitive-motor interference in a sport-specific game-like situation. So, Dai et al. (2017) showed in a dual-task paradigm including a counting (cognitive) task and a jumping-performance task that cognitive-motor interference resulted in decrements in landing as well as jumping performance.

The aim of the study was to examine how visual information-processing task affect motor-performance in a game-like sport-specific situation in elite volleyball experts. We hypothesized that motor performance would decrease in a game-like dual-task situation due to limited and overlapping resources for perceptual-cognitive processing and motor control. Depending on the complexity of the task, we expected a higher motor-cognitive interference in a perceptual-cognitive dual-task (video, dual-task high) than in an only perceptual dual-task (picture, dual-task low).

## Materials and Methods

### Participants

Twenty-four competitive (beach) volleyball players on international and national top level participated in this study. They were players from first to third division in Germany or members of the highest national beach tour; they had elite, partly junior, status, or were part of the national volleyball team. All subjects had ball practice at least four times up to eight times a week during the study. The age ranged from 14 to 30 years (*M* = 19.2 years; *SD* = 4.2) and three of them were male. The athletes were recruited from a German volleyball talent-development center, a first-league volleyball club and other higher-league volleyball clubs from indoor and beach volleyball.

This study was approved by the local ethics committee and informed consent was obtained from all participants (and their parents/legal guardians) prior to any data collection.

### Experimental Setup

**FIGURE 1:**
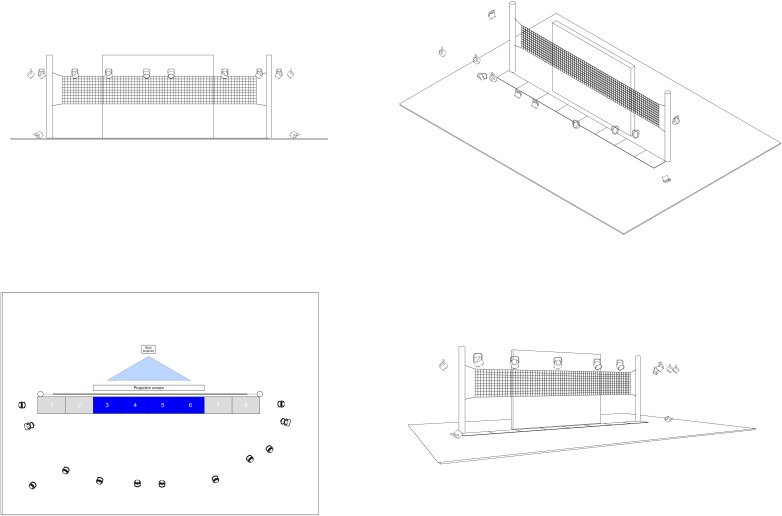
Volleyball setup including net construction, motion capture, force plates, and projection screen.

All measurements were carried out in a motor behavior lab. The test site consisted of a height-adjustable, standard volleyball-net construction (9 m) placed in the middle of the lab. The standard net height for men (2.43 m) and women (2.24 m) was used for testing. To measure volleyball-specific motor-performance parameters (i.e., jumping height and the length of the first step after the ready-block-position), force plates (Kistler^®^) and Qualysis Track Manager (Qualisys^®^ version 2.15) motion-capture system were synchronized and used for each measurement. In total, eight force plates (size: 60 × 80 cm; 1200 Hz) located in series in front of the net construction and 12 QTM Oqus cameras (400 Hz) were set around the net construction (see **Figure 1**).

Additionally, a 5 × 4 m projection screen was positioned parallel (80 cm) to the net construction. The screen was illuminated via a back projector (Optoma EH505 projector). The projector was located 4 m behind the screen to present the stimuli over the whole surface on the screen. For the presentation of the stimuli on the screen, the Neurobehavioral System (NBS) Presentation^®^ software was used and synchronized with QTM and Kistler systems. Before each measurement, the Kistler and Qualysis systems were calibrated.

### Tasks

In this study, a motor performance single-task (i.e., performing isolated block jumps without a second cognitive or perceptual task) and two dual-tasks (performing block jumps plus a perceptual or perceptual-cognitive task) were administered. The setting, starting, and landing area of the players were identical in each task. The starting position was in front of the net, on the middle of force plate number four and five and the landing area was on force plate three (left side) or six (right side).

The following single task and two dual-tasks were implemented:

#### Single Task (ST)

Participants performed self-initiated isolated, maximal block jumps to the right and to the left side in front of the net construction. The screen in front of the net construction was gray and no volleyball field was shown. The instruction was to jump as high as possible.

#### Dual-Task Low (DT-L)

Participants performed self-initiated maximal block jumps to the left and right side while a volleyball-specific image was presented on the 5 × 4 m screen via back projection. The static picture depicted an offensive set play of four opponent players (defense, setter, attackers) from a frontal perspective. A freeze frame at the moment of attacking (i.e., ball-hand contact) was created with a GoPro^®^ Hero. There were two matched pictures with attacker from position II (on the left side from the perspective of the participants) and position IV (on the left side from the perspective of the participants). Participants were positioned in front of the screen and observed the picture from the perspective of an opponent block player. The instruction for the participants was to perform a maximal block jump in front of the attacker at the screen.

#### Dual-Task High (DT-H)

Participants performed maximal block jumps to the right and left side depending on a dynamic perceptual-cognitive load, which consisted of volleyball-specific videos (60 Hz) being presented on the screen via a back projector. The dynamic stimuli were videotaped from a first-person perspective and consisted of four different videos which were created with a GoPro^®^ Hero. (15 Mbit/s; 120 fps) depicting volleyball scenes of offensive set plays with four or five players (defense, setter, attackers). The structure of the offense set play in the videos was always the same: a serve was played at the reception players, a reception was played to the setter, followed by a set play either to position IV or position II and a respective attack from the opponent or outside hitter. The videos were not occluded and ended after the landing of the hitter. Players in the video were recruited from a first-league club (female). The starting positions of all players in the video were standardized and the attackers were instructed to stand still until the start of their attacking approach.

Participants entered the starting position after a “go”-command by the test conductor. Then, they watched the scene from the perspective of an opponent blocking player with the instruction to observe the scene and to perform a maximal blocking action in front of the attacking player (i.e., on the left or right side).

### Procedure

Upon arriving, participants gave informed consent and had an individual and standardized warm-up of 15 min. Then, seven reflective markers were positioned on the back, each big toe, each heel and each hand. To determine the position of the markers in space, a static measurement was conducted. Participants were instructed to stand upright on one of the force plates for 8 s. Upon completing the static measurement, participants started the test session with the three conditions (ST, DT_L, DT_H) in counterbalanced order. In all conditions, participants performed four block jumps with a break of 20 s between each jump and they were reminded before each jump to jump as high as possible.

### Data Analysis and Dependent Measures

Each jump trial was processed in the QTM motion capture system (Version 2.15), exported, and calculated by using MATLAB (MathWorks^®^, Version R2017a). Dependent measure was jumping height. Jumping height was analyzed using the marker at the back of the participants. The vertical distance between the back marker in standing (static measurement) and in the highest point of each jump was calculated with MATLAB (MathWorks^®^, Version R2017a).

As a supplementary measure of motor behavior, we analyzed the length of the first step after ready-block position. The length of the first step was calculated by using the big-toe marker of the foot that made the first step to the right or left side. The distance between the starting position directly before initiating the jump and the first touch on ground was calculated with MATLAB (MathWorks^®^, Version R2017a). All participants used the same volleyball-specific blocking technique (i.e., swing block, which is the preferred technique in elite volleyball), consisting of a three-step approach.

Further parameters were volleyball-specific errors (e.g., net touching) and decision accuracy in all trials and conditions. They were recorded by the experimenter via protocol. An invalid trial in decision accuracy was defined when participants performed a step in the wrong direction.

### Statistical Analyses

Data of each condition and participant were averaged for analysis with Microsoft Excel Version 16.10 and were analyzed with IBM SPSS statistics 25. Repeated-measures ANOVAs with the within-participant factors ST, DT_L, and DT_H were computed to assess differences in the dependent variables jumping performance and the length of the first step after the ready-block-position. Partial eta square was used as a measure of effect size and the level of significance was at *p* < 0.05. Pairwise comparison with Bonferroni correction were used for all *post hoc* tests. Invalid trials were not analyzed.

## Results

Two participants were excluded from all analyses because of too many technique changes between the three conditions.

### Jumping Height

**FIGURE 2:**
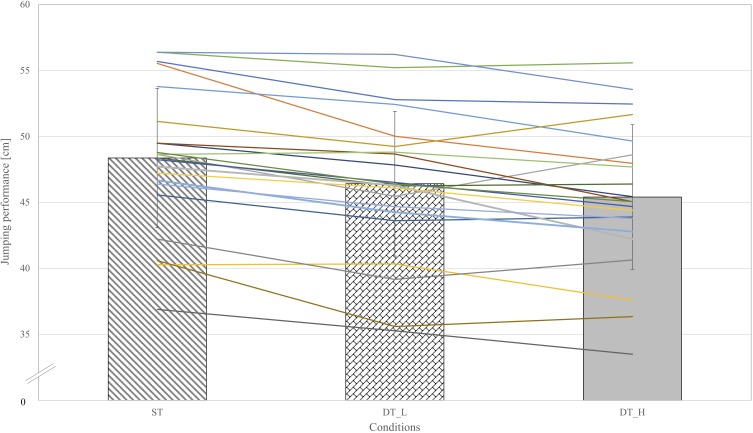
Mean jumping heights over all athletes are presented in the bar graphs (single-task, dual-task low, dual-task high). Individual data of all athletes are in presented in lines.

Mean jumping performance of all included athletes (see **Figure 2**, bar graphs) and individual data of the participants (see **Figure 2**, lines) was calculated based on the individual means of all participants in each condition. Mean jumping height was 48.4 cm (*SD* = 5.3) in ST. In DT_L, mean jumping height was 46.4 cm (*SD* = 5.5) and 45.4 cm (*SD* = 5.5) in DT_H. The results of a repeated-measures ANOVA show a significant effect of conditions on jumping performance *F*(2,42) = 33.64, *p* < 0.001, η_p_^2^ = 0.62. *Post hoc* comparisons reveal that jumping performances in DT_H (*p* < 0.001) and DT_L (*p* < 0.001) were significantly lower than in ST. Between DT_L and DT_H, there was no significant difference (*p* = 0.06).

### Length of the First Step After the Ready-Block-Position

**FIGURE 3:**
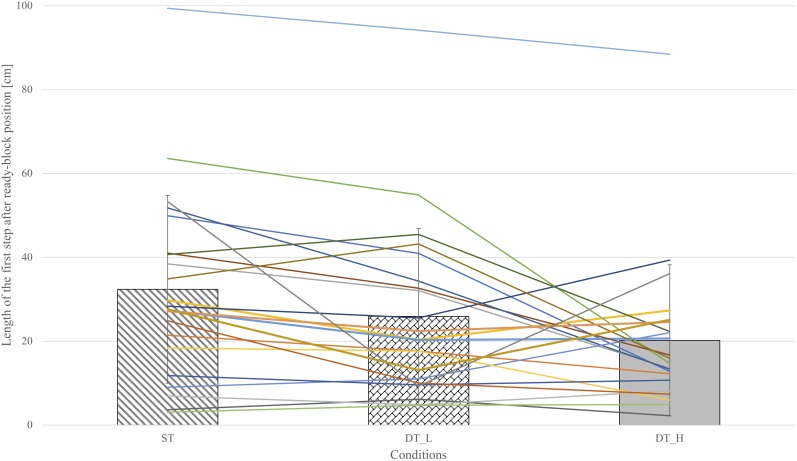
Mean length of the first step after the ready-block-position over all athletes are presented in the bar graphs (single-task, dual-task low, dual-task high). Individual data of all athletes are presented in lines.

The length of the first step after the ready-block-position in the block jumping approach was calculated based on the individual means of all included participants in each condition. **Figure 3** shows the means of the length of the first step after the ready-block-position over all participants in bar graphs. The mean step length in ST was 32.4 cm (*SD* = 22.4), in DT_L 25.9 cm (*SD* = 21.0) and in DT_H 20.2 cm (*SD* = 18.0). The individual data of all athletes are presented as lines in **Figure 3**. The results of the repeated-measures ANOVA shows a significant effect of conditions on the length of the first step after the ready-block-position, *F*(2,42) = 7.90, *p* = 0.001, η_p_^2^ = 0.27. *Post hoc* comparisons reveal that step length in ST was significantly longer than in DT_L (*p* = 0.028) and DT_H (*p* = 0.005). Between DT_H and DT_L, there was no significant difference (*p* = 0.33).

### Further Parameters

The error rate of included athletes in decision accuracy (i.e., the incongruence between ball direction and direction of motor response) was 5.3% in DT_H. The volleyball-specific errors (e.g., net touching) amounted to 1.8% in ST; 5.3% in DT_L and 6.2% in DT_H.

## Discussion

The aim of the present study was to investigate the performance effects of adding perceptual-cognitive tasks to block jumps with a step approach in a dual-task design. Based on the assumption of time and resource sharing between motor and visual-cognitive processing, we expected a visual (DT_L, dual-task low) and a visual-cognitive (DT_H, dual-task high) task to perturb jump-approaching step length as well as jumping performance compared to single-task block jumping (ST). In accordance to our hypothesis, results show that motor-performance (i.e., jumping height) and motor-execution (i.e., length of the first step after the ready-block-position) parameters decreased when secondary visual-cognitive tasks are added. Jumping heights in the perceptual dual-task condition (static picture, DT_L) and also in the perceptual-cognitive dual-task condition (video, DT_H) were significantly lower compared to ST. Contrary to our expectation, it seems that the complexity of the second task had no effect. The prediction that adding visually based decision-making to block jumping would even further detriment performance can, however, not be corroborated.

For an analysis of the approach steps to block jumping, the length of first step after ready-block position was analyzed as a supplementary measure of motor behavior. The first step after ready-block position was significantly lower in DT_H and DT_L than in ST. Again, differential effects between DT_H and DT_L cannot be revealed.

These findings are in line with previous studies that found dual-task costs in motor measures when combined with perceptual-cognitive tasks (Beauchet et al., 2005; Ruffieux et al., 2015). Many studies used an overlearned primary motor task such as walking that is presumed to be executed with little cognitive effort (e.g., the studies by Beurskens et al., 2016b,c). Cognitive-motor interference would show in, e.g., reduced gait velocity or shorter stride length. Beurskens et al. (2016c) demonstrated that a perceptual-cognitive task such as “serial subtraction” reduced walking performance. Also, Plummer-D’Amato et al. (2011) found reduced walking speed while executing a spontaneous speech test in younger and older adults. They hypothesized that walking as the motor task also requires visual processing (e.g., optic flow, visual cues for balance control, etc.), increasing the likelihood of interference between the tasks. The role of visual processing in conceptual ideas for multiple resources and for prediction of interference has already been highlighted by Wickens (2002). Based on this theory, the decrements of motor performance might be explained by an overlap between visual-processing demands for the dual-tasks.

On the basis of a motor-skills taxonomy (Gentile, 1987), walking or approaching are characterized by body transport. In the proposed dimension “action function,” the function of the action is to move the body to a specific location in an allocentric frame. In addition, the environmental context of the task DT_H used here is characterized by in-motion with inter-trial variability, i.e., the conditions are different from one trial to another, as, e.g., the ball’s path and speed changes for each trial. Based on our data in DT_H and DT_L, the effect of additional cost via dynamic environments and online decision-making seems small (i.e., no differences between DT_H and DT_L), but the costs of adding visual-processing requirements induces a strong impact on motor behavior (i.e., DT_L and DT_H differ significantly from ST in jumping height and in length of the first step after the ready-block-position).

In this study, we could not find differential effects between the two secondary tasks (i.e., DT_L and DT_H). Bock (2008) showed higher interference of visually demanding tasks compared to memorization or recall tasks for walking. Similarly, Beurskens and Bock (2013) showed higher interference between visually based tasks compared to a verbal-fluency task (i.e., spelling alphabet) and postulated that two tasks with the need for visual processing overstrain shared resources. The conclusion is, therefore, that increasing the load of visual processing induces interference in a body-transport task, but that the costs of adding on-trial visually based decision-making concerning the direction of the blocking action are not evident or negligible in a sample of elite athletes that are highly familiar with both tasks. Furthermore, some practice conditions might have a greater potential to reduce cognitive-motor interference (e. g., dual-task costs) than others (Strobach et al., 2013). Another option that needs more investigation but could not be tested in this study, is the hypothesis that the unaffected athletes would exhibit higher levels of sport expertise in relation to some expert indicators (e.g., years of experiences at international level, sustained success in major international, globally recognized competition, see Swann et al., 2015 or classifying experts’ performance on based on a special taxonomy, see Baker et al., 2015). A *post hoc* glance on the individual data of athletes, ranging in age from 14 to 30, suggests that the jumping height in some national team athletes decreased less (e.g., no or only little differences in DT_L or/and in DT_H, see **Figures 2**, **3**). Whether this holds in an adequate sample, may need further and specific exploration in the future.

## Conclusion

As seen in the review of Zentgraf et al. (2017), this is one of the first studies which investigated interference effects in game-like situations in elite-sport athletes from the national top in their age range. Our results reveal significant decrements in jumping height and in the length of the first step after ready-block position in a sport-specific dual-task situation. This indicated cognitive-motor interference in a highly automated volleyball-specific task in elite athletes. In elite sports, it is essential to tap the full physical potential also in a game situation. However, even overlearned and highly repeated motor performance in elite athletes (i.e., jumping height and length of the first step after the ready-block-position) decreased under secondary visually based tasks. In this vein, it is necessary to analyze sport-specific attentional demands and to investigate whether and how perceptual-cognitive skills might be practiced in a sport-specific way (Zentgraf et al., 2017) to minimize cognitive-motor interference and improve transfer to performance in competition. This will be the focus of upcoming studies.

## Author Contributions

M-TF prepared the setup together with KZ, collected the data from the participants, analyzed the data, and wrote the manuscript. KZ was grant applicant, developed the research design, supported setup preparation, checked the data, and wrote the manuscript together with M-TF.

## Conflict of Interest Statement

The authors declare that the research was conducted in the absence of any commercial or financial relationships that could be construed as a potential conflict of interest.
